# Immunological and prognostic significance of tumour necrosis in colorectal cancer

**DOI:** 10.1038/s41416-023-02258-2

**Published:** 2023-04-08

**Authors:** Meeri Kastinen, Päivi Sirniö, Hanna Elomaa, Maarit Ahtiainen, Sara A. Väyrynen, Karl-Heinz Herzig, Sanna Meriläinen, Raila Aro, Reetta Häivälä, Tero Rautio, Juha Saarnio, Erkki-Ville Wirta, Olli Helminen, Toni T. Seppälä, Teijo Kuopio, Jan Böhm, Anne Tuomisto, Jukka-Pekka Mecklin, Markus J. Mäkinen, Juha P. Väyrynen

**Affiliations:** 1 Translational Medicine Research Unit, Medical Research Center Oulu, Oulu University Hospital, and University of Oulu, Oulu, Finland; 2 Department of Biological and Environmental Science, University of Jyväskylä, Jyväskylä, Finland; 3 Department of Education and Research, Wellbeing services county of Central Finland, Jyväskylä, Finland; 4 Department of Pathology, Wellbeing services county of Central Finland, Jyväskylä, Finland; 5 Department of Internal Medicine, Oulu University Hospital, Oulu, Finland; 6 Research Unit of Biomedicine, Medical Research Center Oulu, University of Oulu, Oulu University Hospital, Oulu, Finland; 7 Department of Pediatric Gastroenterology and Metabolism, Poznan University of Medical Sciences, Poznan, Poland; 8 Department of Gastroenterology and Alimentary Tract Surgery, Tampere University Hospital, Tampere, Finland; 9 Faculty of Medicine and Health Technology, Tampere University and Tays Cancer Centre, Tampere University Hospital, Tampere, Finland; 10 Department of Gastrointestinal Surgery, Helsinki University Central Hospital, University of Helsinki, Helsinki, Finland; 11 Applied Tumor Genomics, Research Program Unit, University of Helsinki, Helsinki, Finland; 12 Faculty of Sport and Health Sciences, University of Jyväskylä, Jyväskylä, Finland

**Keywords:** Colorectal cancer, Cancer microenvironment, Tumour biomarkers

## Abstract

**Background:**

Colorectal cancer (CRC) causes the second most cancer deaths worldwide, but the disease course varies according to tumour characteristics and immunological factors. Our objective was to examine the associations of tumour necrosis with tumour characteristics, immune cell infiltrates, serum cytokine concentrations, as well as prognosis in CRC.

**Methods:**

Three independent CRC cohorts, including 1413 patients, were analysed. Associations of the areal percentage of tumour necrosis with clinicopathologic parameters, tumour infiltrating immune cells, cytokine concentrations in systemic and mesenteric vein blood, and survival were examined.

**Results:**

Higher tumour necrosis percentage associated with shorter colorectal cancer-specific survival independent of tumour grade, T, N or M-class, mismatch repair status, *BRAF* status, and other possible confounding factors. In the largest cohort (*N* = 1100), the HR for high tumour necrosis percentage (≥40% vs. <3%) was 3.22 (95% CI 1.68–6.17, *P*_trend_ < 0.0001). Tumour necrosis percentage positively correlated with peripheral serum levels of CXCL8, a proinflammatory chemokine, and negatively correlated with mesenteric serum levels of CXCL10 and mast cell densities in the invasive margin of the tumour.

**Conclusions:**

Our results support the value of tumour necrosis as a prognostic factor in colorectal cancer. CXCL8 may have a role in the systemic effects of tumour necrosis.

## Background

Colorectal cancer (CRC) is the third most common cancer in the world, and it causes the second most cancer deaths worldwide [1]. The prognosis of CRC is mainly based on the TNM (tumour, node, metastasis) classification evaluating disease extent, while additional prognostic or predictive factors include tumour grade, lymphovascular or perineural invasion, tumour budding, and mismatch repair (MMR), *BRAF*, and *RAS* mutation status [2]. However, these factors still incompletely capture the heterogeneity of tumour behaviour, and new prognostic factors are much needed.

Colorectal cancers frequently contain some amounts of necrosis, and tumour necrosis is a negative prognostic factor in CRC [3]. The local inflammatory response has been thought to be weaker in tumours with extensive necrosis [4], but few studies have systematically assessed this. The local anti-tumour immune response is a favourable prognostic marker in CRC which can be reflected by tumour infiltrating lymphocytes and Crohn’s like immune reaction [4]. Tumour necrosis may restrain this beneficial immune reaction thorough necrosis-induced systemic inflammation [5]. In addition, tumour necrosis has been hypothesised to increase the secretion of inflammatory factors and participate in the modulation of the tumour microenvironment [3, 5], but the more detailed mechanisms have not yet been established. Moreover, the associations between tumour necrosis and specific circulating cytokines in CRC are still unclear.

The aim of this study was to (a) evaluate the independent prognostic role of tumour necrosis in CRC, and to (b) investigate the local and systemic alterations of immune and inflammation-related biomarkers associated with tumour necrosis. We analysed three different cohorts: Cohort 1 consisted of 1100 CRC patients with comprehensive clinicopathologic annotations and long follow-up for survival analyses; Cohort 2 consisted of 287 patients, from whom more detailed immune cell types and peripheral cytokine levels were analysed; and Cohort 3 consisted of 26 patients that had peripheral and mesenteric venous blood samples analyzed in a unique study setup, enabling more direct analysis of the tumour-derived factors entering the circulation. Our focus was to clarify the reaction patterns (cytokine secretion and immune cell infiltration into tumours) tumour necrosis elicits in the host, as well as their impact on the disease course.

## Methods

### Patients

**Fig. 1:**
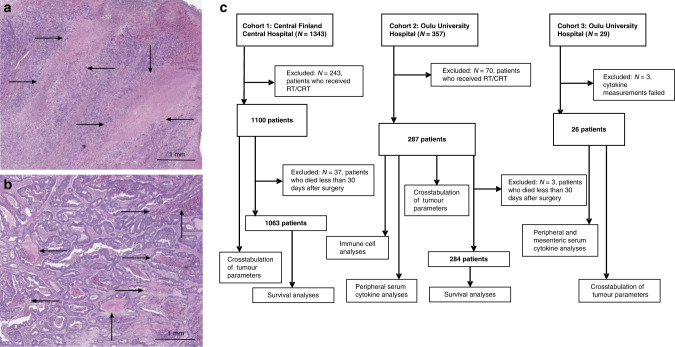
Tumour necrosis in colorectal cancer. **a** Large regions of coagulative necrosis in a haematoxylin & eosin-stained tumour resection specimen. **b** Several smaller pools of intraluminal necrosis in another resection sample. **c** Flowcharts of the patients and cohorts analyzed in the study. RT/CRT radiotherapy or chemoradiotherapy.

Three independent cohorts were analyzed (Fig. 1).

Cohort 1 was retrospectively collected in Central Finland Central Hospital in Jyväskylä, consisting of 1343 patients who had undergone tumour resection during 2000–2015 and from whom adequate tumour samples were available [6, 7]. Cohort 2 was a previously described, prospectively collected cohort of colorectal cancer patients (*N* = 357) operated on at Oulu University Hospital between 2006 and 2014 [8, 9].

Patients who had received preoperative radiotherapy or chemoradiotherapy (Cohort 1: *N* = 243; Cohort 2: *N* = 70) were excluded from further analyses, and patients who died in less than 30 days after surgery (Cohort 1: *N* = 37; Cohort 2: *N* = 3) were excluded from survival analyses, thus data of 1063 or 1100 patients from Cohort 1 and data of 284 or 287 patients from Cohort 2 could be utilised. Clinical endpoints were cancer-specific survival (CSS), defined as the time from surgery to cancer death or to the end of follow-up and overall survival (OS) defined as the time from surgery to death or to the end of follow-up. The median follow-up time for censored cases was 10 years (Cohort 1: IQR 7.3–10 years, Cohort 2: IQR 8.6–10 years). In addition of offering validation for survival analysis, Cohort 2 provided information about the tumour immune cell profile and cytokine levels in the systemic circulation.

Cohort 3 consisted of 29 colorectal cancer patients who were operated on in Oulu University Hospital during 2020–2021. Blood samples from the mesenteric vein were drawn during the operation and stored for analysis. Peripheral venous blood samples were also collected. Three patients were excluded because of the inadequate sample for cytokine analysis, leaving us a total of 26 cases.

### Serum samples and analyses

For Cohorts 2 and 3, preoperative serum samples were collected and stored at −70 °C until the analysis. For Cohort 3, the operating surgeons collected an additional serum sample from the mesenteric vein during the operation. For Cohort 2 patients operated between 2006 and January 2010, Bio-Plex Human pre-manufactured 27-Plex Cytokine panel (Bio-Rad) was used to measure serum cytokine levels [10]. 14 cytokines had values outside the assay working range and were therefore excluded [10]. For Cohort 2 patients operated between February 2010 and 2014 and Cohort 3 patients, Olink Target 96 Immuno-Oncology Panel was used to assess cytokine concentrations [11], and the nine cytokines overlapping with the 13 analyzed with the Bio-Plex were considered for the inclusion in this study.

In addition, IL6 was excluded from the Olink results, as 50 of the measurements in Cohort 2 (2010–2014) and 15 in Cohort 3, were outside the assay working range or did not meet quality control criteria. For Cohort 3 patients, additional peripheral/mesenteric cytokine concentration difference values were calculated by subtracting peripheral vein serum values from mesenteric vein serum values, to measure the difference in cytokine levels between systemic blood and mesenteric vein blood.

### Histopathological analyses

Haematoxylin & eosin staining was used in analysing the tumour specimens that were fixed in 10% formalin and then embedded in paraffin. TNM stage was determined by the UICC/AJCC (Union for International Cancer Control/The American Joint Committee on Cancer) criteria and grade according to the WHO criteria. The Glasgow Microenvironment score (GMS) was evaluated [12], composed of Klintrup–Mäkinen score [13] and tumour stroma percentage [14]. In accordance with a previous study, tumour necrosis in hematoxylin & eosin-stained sections was identified as an area with increased eosinophilia and nuclear pyknosis, karyorrhexis, and karyolysis, i.e., nuclear shrinkage, fragmentation and disappearance, with shadows of tumour cells visible to the variable extent [3]. The average percentage of tumour necrosis in all available tumour sections was estimated visually from whole slide images [3]. The evaluation was performed blinded to the study endpoints. For Cohorts 1 and 2, MMR enzyme status and *BRAF* V600E mutation status were assessed using immunohistochemistry as described earlier [6, 15–17].

### Immune cell analyses

The analyses of immune cell densities in tumour samples, based on immunohistochemistry and quantitative image analysis, have been described earlier. For Cohort 1, tumour infiltrating T cells were quantified by using QuPath bioimage analysis software [18]. It used supervised machine learning algorithms to identify CD3 + and CD8 + cells in the invasive margin and centre of tumour [6]. For Cohort 2, computer-assisted, ImageJ-based, analysis method was used to count the densities of immune cells in the invasive margin and centre of tumour [9, 19, 20].

### Statistical analyses

Statistical analyses were carried out using IBM SPSS Statistics for Windows (IBM Corp. version 27.0). We considered findings with two-sided *P* < 0.05 statistically significant.

Crosstabulation was used to investigate the associations between tumour necrosis percentage categories and tumour and patient characteristics. The tumour necrosis percentage was categorised into four classes <3%, 3–9.9%, 10–39.9% and ≥40%, in accordance with a previous study [3]. We used Pearson’s correlation analysis to examine the correlations of tumour necrosis percentage (continuous) with cytokines and tumour infiltrating immune cells. Linear regression models were used to adjust the associations for pre-determined covariates. As covariates in Cohorts 1 and 2, we used age (continuous), sex (male, female), MMR enzyme status (proficient, deficient), *BRAF* V600E mutation status (wild-type, mutant), tumour location (colon, rectum), and stage (I–II, III–IV). In Cohort 3, only age (continuous) and Stage (I–II, III) were included, considering the lower sample size. The continuous variables with positive skewness were logarithmically transformed. The linearity, normality, and homoscedasticity assumptions were checked by using normal probability plot of residuals and scatterplots of residuals compared to predicted values. The statistical significance of the associations between MMR status and immune cells were determined by using the Mann–Whitney *U* test.

Cox regression models and Kaplan–Meier estimates were used to assess the associations of tumour necrosis categories with cancer-specific and overall survival. The assumptions for Cox regression model were checked by inspecting Schoenfeld residual plots that supported the proportionality of hazards during the 10-year follow-up. Multivariable Cox regression models included the following pre-determined covariates: age (<65, 65–75, >75), sex (male, female), T (1–2, 3–4), N (0, 1–2), M (0, 1), MMR enzyme status (proficient, deficient), *BRAF* V600E mutation status (wild-type, mutant), tumour location (proximal colon, distal colon, rectum), time of operation (Cohort 1: 2000–2005, 2006–2010, 2011–2015; Cohort 2: 2006- Jan. 2010, Feb. 2010–2014), lymphatic or venous invasion (no, yes), and grade (low-grade, high grade). We excluded patients who died within 30 days of having surgery (Cohort 1: *N* = 37; Cohort 2: *N* = 3).

## Results

### Necrosis in relation to tumour characteristics and survival

We first analyzed the associations of tumour necrosis with tumour and patient characteristics in 1100 patients of Cohort 1 (Table 1). The strongest associations of tumour necrosis percentage were observed with tumour invasion depth (T class) (*P* < 0.0001), tumour location in the distal colon (*P* = 0.0001), high Glasgow Microenvironment Score (GMS) (*P* < 0.0001) and high disease stage (*P* < 0.0001). High tumour grade (*P* = 0.0003), and the presence of nodal (*P* = 0.0002) and distant metastases (*P* = 0.042) were also associated with high tumour necrosis percentage. MMR deficiency and *BRAF* V600E mutation were associated with low tumour necrosis percentage (*P* = 0.004 and 0 = 0.017, respectively). Overall, high tumour necrosis percentage appeared to be associated with several adverse tumour characteristics.

**Table 1:** Tumour and patient characteristics in Cohort 1 and their association with tumour necrosis.

Characteristic		Total *N* (%)	Tumour necrosis percentage				
			<3%	3–9.9%	10–39.9%	≥40%	*P* value
All cases		1100 (100%)	104 (9.5%)	593 (54%)	341 (31%)	62 (5.6%)	
Sex	Male	557 (51%)	51 (9.2%)	304 (55%)	171 (31%)	31 (5.6%)	0.97
	Female	543 (49%)	53 (9.8%)	289 (53%)	170 (31%)	31 (5.7%)	
Age	<65	290 (26%)	23 (7.9%)	149 (51%)	99 (34%)	19 (6.6%)	0.58
	65–75	381 (35%)	35 (9.2%)	215 (56%)	109 (29%)	22 (5.8%)	
	>75	429 (39%)	46 (11%)	229 (53%)	133 (31%)	21 (4.9%)	
Tumour location	Proximal colon	536 (49%)	63 (12%)	284 (53%)	158 (29%)	31 (5.8%)	0.0001
	Distal colon	404 (37%)	24 (5.9%)	205 (51%)	146 (36%)	29 (7.1%)	
	Rectum	160 (15%)	17 (11%)	104 (65%)	37 (23%)	2 (1.3%)	
T	T1	58 (5.3%)	13 (22%)	34 (59%)	11 (19%)	0 (0%)	<0.0001
	T2	169 (15%)	24 (14%)	108 (64%)	37 (22%)	0 (0%)	
	T3	677 (62%)	52 (7.7%)	370 (55%)	221 (33%)	34 (5%)	
	T4	196 (18%)	15 (7.7%)	81 (41%)	72 (37%)	28 (14%)	
N	N0	626 (57%)	71 (11%)	361 (58%)	171 (27%)	23 (3.7%)	0.0002
	N1	275 (25%)	18 (6.5%)	139 (51%)	95 (35%)	23 (8.4%)	
	N2	199 (18%)	15 (7.5%)	93 (47%)	75 (38%)	16 (8.0%)	
M	M0	947 (86%)	93 (9.8%)	519 (55%)	288 (30%)	47 (5.0%)	0.042
	M1	153 (14%)	11 (7.2%)	74 (48%)	53 (35%)	15 (9.8%)	
Stage	I	184 (17%)	29 (16%)	119 (65%)	36 (20%)	0 (0%)	<0.0001
	II	408 (37%)	40 (9.8%)	219 (54%)	126 (31%)	23 (5.6%)	
	III	355 (32%)	24 (6.8%)	181 (51%)	126 (35%)	24 (6.8%)	
	IV	153 (14%)	11 (7.2%)	74 (48%)	53 (35%)	15 (9.8%)	
WHO grade	Low grade	903 (82%)	78 (8.6%)	501 (55%)	284 (31%)	40 (4.4%)	0.0003
	High grade	197 (18%)	26 (13%)	92 (47%)	57 (29%)	22 (11%)	
Glasgow Microenvironment Score	GMS0	469 (43%)	52 (11%)	288 (61%)	119 (25%)	10 (2.1%)	<0.0001
	GMS1	329 (30%)	37 (11%)	163 (50%)	106 (32%)	23 (7.0%)	
	GMS2	302 (27%)	15 (5.0%)	142 (47%)	116 (38%)	29 (9.6%)	
MMR enzyme status	Proficient	931 (85%)	77 (8.3%)	513 (55%)	293 (31%)	48 (5.2%)	0.004
	Deficient	169 (15%)	27 (16%)	80 (49%)	48 (28%)	14 (8.3%)	
BRAF status^a^	Wild-type	916 (83%)	76 (8.3%)	497 (54%)	293 (32%)	50 (5.5%)	0.017
	Mutant	182 (17%)	28 (15%)	94 (52%)	48 (26%)	12 (6.6%)	

^a^Data missing from two patients.

**Fig. 2:**
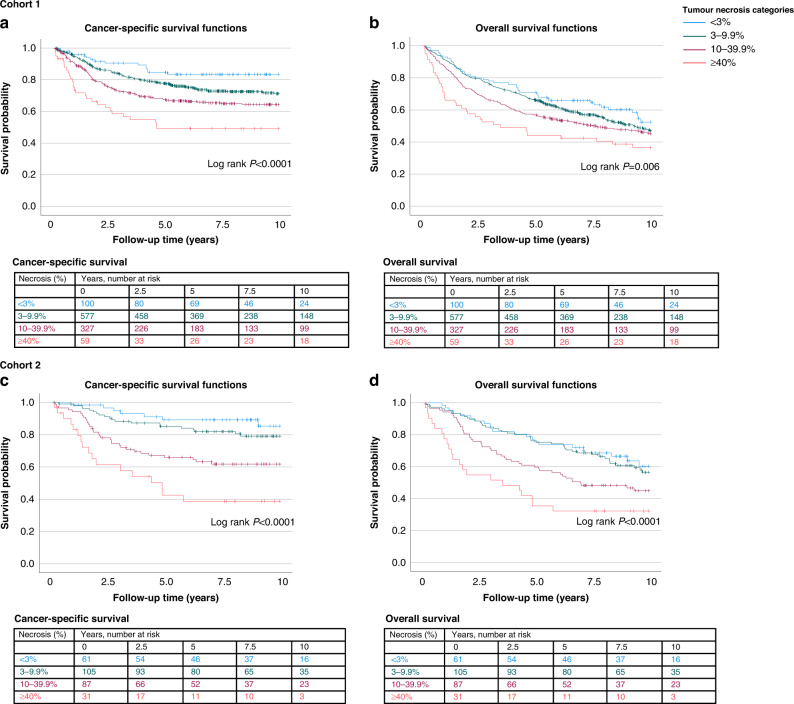
Kaplan–Meier survival analyses. The associations of tumour necrosis with (**a**) cancer-specific survival (CSS) and (**b**) overall survival (OS) in Cohort 1. The associations of tumour necrosis with (**c**) CSS and (**d**) OS in Cohort 2.

In 10-year survival analyses, there were 530 (49.9%) deaths of which 295 (27.8%) were cancer deaths. Kaplan–Meier survival functions showed a significant association between tumour necrosis categories and survival, especially in the CSS analysis (log-rank *P* < 0.0001) (Fig. 2). In accordance with our hypothesis, high tumour necrosis percentage was associated with shorter CSS and OS. Cox regression analyses showed that the association of high tumour necrosis percentage with shorter CSS and OS was independent of tumour grade, T, N and M-class, MMR status, *BRAF* status and other potential confounding factors (Table 2 and Supplementary Table S1). In multivariable analysis, the hazard ratio (HR) for patients with ≥40% necrotic area (vs. <3%) was 3.22 (95% CI 1.68–6.17, *P*_trend_ < 0.0001).

**Table 2:** Cox regression models for the associations between tumour necrosis percentage and survival in Cohorts 1 and 2.

		Cancer-specific survival			Overall survival		
Tumour necrosis percentage	No. of cases	No. of events	Univariable HR (95% CI)	Multivariable HR (95% CI)	No. of events	Univariable HR (95% CI)	Multivariable HR (95% CI)
Cohort 1							
<3%	100	15	1 (referent)	1 (referent)	42	1 (referent)	1 (referent)
3–9.9%	577	145	1.73 (1.01–2.94)	1.76 (1.02–3.04)	278	1.19 (0.87–1.65)	1.19 (0.85–1.66)
10–39.9%	327	107	2.45 (1.43–4.20)	2.35 (1.34–4.11)	173	1.41 (1.01–1.97)	1.44 (1.02–2.04)
≥40%	59	28	4.24 (2.27–7.94)	3.22 (1.68–6.17)	37	1.96 (1.26–3.05)	1.88 (1.19–2.97)
* P*_trend_			<0.0001	<0.0001		0.0010	0.0011
Cohort 2							
<3%	61	7	1 (referent)	1 (referent)	22	1 (referent)	1 (referent)
3–9.9%	105	20	1.67 (0.71–3.94)	1.80 (0.71–4.60)	43	1.13 (0.68–1.89)	1.08 (0.62–1.88)
10–39.9%	87	32	3.70 (1.63–8.39)	1.85 (0.75–4.55)	47	1.78 (1.07–2.94)	1.10 (0.62–1.96)
≥40%	31	17	7.53 (3.12–18.18)	3.39 (1.28–8.96)	21	3.14 (1.72–5.71)	1.70 (0.86–3.36)
* P*_trend_			<0.0001	0.018		<0.0001	0.19

*HR* hazard ratio, *CI* confidence interval.

Multivariable Cox regression models were adjusted for age (<65, 65–75, >75), sex (male, female), T (1–2, 3–4), N (0, 1–2), M (0, 1), tumour location (proximal colon, distal colon, rectum), year of operation (Cohort 1: 2000–2005, 2006–2010, 2011–2015; Cohort 2: 2006- Jan. 2010, Feb. 2010–2014), lymphatic or venous invasion (no, yes), grade (low-grade, high grade), MMR status (proficient, deficient), and *BRAF* status (wild-type, mutant). We excluded patients who died 30 days or less after having surgery (*N* = 37, in Cohort 1 and *N* = 3 in Cohort 2). *P*_trend_ values were calculated by using the four ordinal categories of tumour necrosis percentage as a continuous variable in univariable and multivariable Cox proportional hazard regression models.

We then investigated how the prognostic value of tumour necrosis percentage compares with that of the Glasgow Microenvironment score (GMS) [12] (Supplementary Table S2), which is a recently introduced prognostic tool in CRC that combines peritumoral inflammation (Klintrup–Mäkinen score) [13] and tumour stroma percentage [14]. These analyses indicated that high tumour necrosis percentage and high GMS were independent predictors of shorter CSS.

For Cohort 1, CD3 + and CD8 + T cell density data were available, and high tumour necrosis percentage showed a weak negative correlation with CD3 + T cell density in the centre of the tumour (beta = −0.099, *P* = 0.0013) independent of disease stage, MMR status, and other tumour and patient characteristics (Supplementary Table S3).

Next, we sought to validate the results from Cohort 1 and further study the associations of tumour necrosis percentage with tumour infiltrating immune cells and serum cytokines in an independent cohort of 287 CRC patients (Cohort 2).

In the validation cohort, mainly similar associations were seen between tumour necrosis percentage and clinicopathologic features, as in Cohort 1 (Supplementary Table S4): High tumour necrosis percentage was associated with an advanced TNM stage (*P* < 0.0001), high grade (*P* < 0.0001) and MMR proficiency (*P* < 0.0001). There was more statistical power in Cohort 1 due to the higher numbers, which might explain why parameters like tumour location and *BRAF* mutation did not show statistically significant associations in Cohort 2.

In 10-year survival analyses, there were 133 (46.8%) deaths and 76 (26.8%) cancer deaths in Cohort 2. As in Cohort 1, high tumour necrosis percentage was associated with shorter CSS and OS in univariable analysis (*P* < 0.0001 for both) (Table 2 and Fig. 2). In multivariable analysis, high tumour necrosis percentage was independently associated with shorter CSS (HR for ≥ 40% vs. <3% 3.39, 95% CI 1.28–8.96, *P*_trend_ = 0.018) (Table 2 and Supplementary Table S5).

We analyzed the correlations between tumour necrosis percentage and an expanded group of immune cell types in tumours (Supplementary Table S6). There was no statistically significant correlation between tumour necrosis percentage and CD3 + T cell density in the centre of tumour (multivariable beta = −0.071, *P* = 0.226). Instead, mast cell densities in the invasive margin were negatively correlated with tumour necrosis percentage (multivariable beta = −0.196, *P* = 0.001), while CD3 + , CD8 + or FOXP3 + T cells, and neutrophils did not show significant correlations in the multivariable models. We also analyzed immune cell densities in relation to MMR status. The number of immune cells was higher in tumours that were MMR-deficient (Supplementary Table S7).

We then aimed to characterise the associations between tumour necrosis percentage and systemic inflammatory markers (Table 3). For these analyses, Cohort 2 was subdivided into patients operated in 2006–Jan. 2010 (Cohort 2A) and Feb. 2010–2014 (Cohort 2B), based on the serum cytokine assay that was used. CXCL8, a proinflammatory chemokine that is also known as interleukin 8 (IL8), showed a positive correlation with tumour necrosis in both Cohorts 2A and 2B (beta = 0.206 and 0.209, *P* = 0.009 and 0.035, respectively). Of other cytokines, tumour necrosis percentage positively correlated with IL7 in Cohort 2B but not in 2 A (beta = 0.190 and 0.083, *P* = 0.017 and 0.375, respectively), and with IL6 in Cohort 2A (beta = 0.191, *P* = 0.043).

**Table 3:** Correlations between tumour necrosis percentage and serum cytokine levels.

	Cohort 2A (2006–January 2010)					Cohort 2B (February 2010–2014)				
Variable	*N* (unadjusted, adjusted)	Unadjusted		Adjusted		*N* (unadjusted, adjusted)	Unadjusted		Adjusted	
		Pearson *r*	*P* value	Beta	*P* value		Pearson *r*	*P* value	Beta	*P* value
IL1RN	117, 116	0.091	0.33	−0.003	0.97	–^a^	–	–	–	–
IL4	117, 116	0.073	0.43	0.063	0.50	–^a^	–	–	–	–
IL6	117, 116	0.245	0.008	0.191	0.043	–^b^	–	–	–	–
IL7	117, 116	0.121	0.19	0.083	0.38	150, 150	0.242	0.003	0.190	0.017
CXCL8	117, 116	0.259	0.005	0.206	0.035	150, 150	0.212	0.009	0.209	0.009
IL9	117, 116	0.010	0.91	0.019	0.84	–^a^	–	–	–	–
IL12	117, 116	−0.013	0.89	−0.046	0.62	150, 150	−0.114	0.166	−0.137	0.095
IFNG	117, 116	0.088	0.34	0.087	0.35	150, 150	0.001	0.990	0.049	0.54
CXCL10	117, 116	0.043	0.65	0.096	0.32	150, 150	−0.085	0.302	−0.083	0.30
CCL4	117, 116	0.023	0.81	0.020	0.83	150, 150	0.100	0.221	0.083	0.29
CCL2	117, 116	0.116	0.21	0.110	0.24	150, 150	−0.025	0.764	−0.023	0.77
CCL11	117, 116	−0.045	0.63	0.025	0.79	–^a^	–	–	–	–
PDGFB	117, 116	0.117	0.21	0.101	0.28	150, 150	−0.005	0.953	−0.019	0.81

^a^Not included in this assay.

^b^Excluded from the analysis, as 50 measurements were outside the assay working range or did not meet quality control criteria.

The adjusted correlation coefficients (Beta) were based on multivariable linear regression models that included age (continuous), sex (male, female), tumour location (colon, rectum), Stage (I–II, III–IV), MMR status (proficient, deficient) and *BRAF* status (wild-type, mutant).

### Necrosis in relation to cytokine concentrations in mesenteric vein blood

Finally, we utilised a unique study setup of mesenteric venous blood sampling during operation from 26 patients to evaluate the associations of tumour necrosis with circulating inflammatory mediators near the tumour site. Patient characteristics are presented in Supplementary Table S8. As Cohort 2 analyses suggested that peripheral serum CXCL8 levels were positively correlated with tumour necrosis percentage, we sought to validate this (Table 4). Although the effect size was comparable to that seen in Cohort 2 (beta = 0.159), statistical significance was not reached (*P* = 0.465), related to the smaller sample size. CXCL8 concentrations were frequently higher in the mesenteric venous blood compared to the peripheral blood, and the positive correlation between tumour necrosis and mesenteric serum CXCL8 (beta = 0.242, *P* = 0.325) was stronger than with peripheral CXCL8. The CXCL8 difference variable (mesenteric-peripheral) also showed a tendency towards a positive correlation with tumour necrosis percentage (beta = 0.144, *P* = 0.499).

**Table 4:** Correlations between tumour necrosis percentage and peripheral and mesenteric vein serum cytokine concentrations.

Variable	*N*	Peripheral cytokines				Mesenteric cytokines				Difference (mesenteric-peripheral)			
		Unadjusted		Adjusted		Unadjusted		Adjusted		Unadjusted		Adjusted	
		Pearson *r*	*P* value	Beta	*P* value	Pearson *r*	*P* value	Beta	*P* value	Pearson *r*	*P* value	Beta	*P* value
IL7	26	−0.015	0.94	−0.032	0.88	0.041	0.84	0.081	0.71	−0.039	0.85	−0.002	0.99
CXCL8	26	0.219	0.28	0.159	0.47	0.297	0.14	0.242	0.33	0.152	0.46	0.144	0.50
IL12	26	−0.123	0.55	−0.251	0.27	−0.153	0.46	−0.258	0.24	0.109	0.60	0.126	0.55
IFNG	26	−0.273	0.18	−0.228	0.30	−0.393	0.047	−0.363	0.076	−0.068	0.74	−0.091	0.67
CXCL10	26	−0.203	0.32	−0.466	0.049	−0.386	0.051	−0.460	0.022	0.111	0.59	0.255	0.27
CCL4	26	0.035	0.87	−0.027	0.90	−0.068	0.74	−0.087	0.68	−0.121	0.56	−0.086	0.69
CCL2	26	0.091	0.66	0.042	0.85	0.189	0.36	0.162	0.55	0.162	0.43	0.257	0.29
PDGFB	26	−0.040	0.85	−0.082	0.70	0.160	0.44	0.078	0.73	0.093	0.65	0.096	0.65

The adjusted correlation coefficients (Beta) were based on multivariable linear regression models that included age (continuous) and Stage (I–II and III). The difference variable was calculated by subtracting the peripheral vein serum value from the mesenteric.

Of the other cytokines, tumour necrosis percentage negatively correlated with CXCL10 levels in the mesenteric serum (beta = −0.460, *P* = 0.022), but there were no statistically significant correlations between tumour necrosis percentage and any of the difference (mesenteric-peripheral) variables.

## Discussion

The objectives of this study were to investigate the associations between tumour necrosis, local and systemic immune microenvironment, and prognosis in colorectal cancer. High tumour necrosis percentage was associated with adverse prognostic parameters such as high TNM stage and grade, but also with shorter cancer-specific survival independent of these factors. CXCL8, a proinflammatory chemokine, showed significant positive correlation with tumour necrosis. Mast cells in invasive margin had a negative correlation with tumour necrosis. Despite the small sample size in Cohort 3, CXCL10 levels in the mesenteric serum also had a statistically significant negative correlation with necrosis. These findings highlight the associations of tumour necrosis with the host responses to tumour and the disease course.

Factors influencing the development of tumour necrosis in CRC are not clear. Tumour necrosis in CRC may be due to hypoxic conditions and fast proliferation rate of tumour cells [21–23], but a previous study [3] showed no significant association between tumour necrosis and microvascular density in tumour or tumour cell proliferation rate. Hypoxia is associated with inducing genes that promote stemness of cancer cells [24]. Hypoxic tumours also have been associated with worse survival [25]. Therefore, hypoxia may contribute to shortened survival in necrotic tumours. In our study, high tumour necrosis percentage was associated with several tumour characteristics such as high TNM stage, high grade, and MMR proficient status. Other studies have reported similar findings [3, 22, 23, 26–29]. Apoptotic cell death could be more common in MMR-deficient tumours compared to MMR proficient [30, 31]. Thus, the potential differences in cell death mechanisms might account for the lower necrosis percentage in MMR-deficient tumours. However, more research on this subject is required.

Mast cell densities in the invasive margin of the tumour were found to negatively correlate with tumour necrosis percentage. Mast cells promote angiogenesis, neovascularization, and inflammatory response, and are normally present in the gastrointestinal tract and frequently observed in gastrointestinal cancers [32]. Thus, the negative correlation between mast cells densities and tumour necrosis could be explained by insufficient angiogenesis and neovascularization. The absence of mast cells could promote tumour necrosis. Similarly, in gastric cancer, mast cell counts inversely correlated with necrosis, and this was hypothesised to be explained by the regulation of tumour angiogenesis and neovascularization by mast cells [33]. Mast cells may have both anti- and protumor functions [34], depending on the factors mast cells secrete, tumour type, as well as the location of the mast cell infiltrate in the tumour [34, 35]. Mast cells in the invasive margin were not connected to parameters like TNM stage, liver metastasis, or survival in previous CRC studies [34, 35]. Conversely, in breast cancer, mast cells in the invasive margin associated with less aggressive tumour behaviour [36].

CD3 + T cell densities showed a weak negative correlation with tumour necrosis percentage in Cohort 1, but this was not confirmed in Cohort 2. Tumour necrosis also associated with a weaker lymphocyte infiltration in CRC patients with TNM stage IV without residual disease [26]. We found a negative correlation between tumour necrosis percentage and CXCL10 concentrations in mesenteric venous serum (beta = −0.460, *P* = 0.022), and a trend towards a negative correlation between tumour necrosis percentage and IFNG (beta = −0.363, *P* = 0.076). These associations may also reflect the suppression of local anti-tumour inflammatory response in necrotic tumours, considering that CXCL10 and IFNG are regarded as important cytokines in the antitumorigenic T helper type 1 response [37]. Further studies are required to evaluate the associations of tumour necrosis with more specific immune cell subtypes such as T helper subsets [4].

Peripheral serum CXCL8 levels were found to have positive correlation with tumour necrosis percentage, and such tendency was also observed between the mesenteric vein serum CXCL8 and tumour necrosis percentage, suggesting that CXCL8 may transmit the protumor signals related to tumour necrosis. Previous studies also support the role of CXCL8 in tumour progression: CXCL8 enhanced tumour proliferation rate, angiogenesis and the ability to metastasize [38, 39]; recruited granulocytes at the site of inflammation [40, 41]; and high concentrations of CXCL8 were connected to worse survival in CRC patients [41]. In endometrial cancer, tumour necrosis was associated with a 3.8-fold change in the expression of *CXCL8* [42]. In hepatocellular carcinoma, CXCL8 upregulation was linked to more aggressive invasion [43]. Inhibition of CXCL8 could be considered a therapeutic target, and the tumour necrosis percentage could be evaluated as a potential predictive biomarker.

Increased systemic inflammation and reduced local inflammation in tumours with abundant necrosis have also been reported in some previous studies [22, 44]. Other proinflammatory cytokines have been shown to have a connection to increased necrosis. In study by Guthrie et al., IL6 showed association to increased tumour necrosis [44]. IL6 is a proinflammatory cytokine and has been associated with tumour progression, proliferation, differentiation, and aggressivity. CXCL8 was not assessed in that study.

We found that high tumour necrosis percentage was associated with shorter cancer-specific survival in two independent cohorts, involving more than 1300 patients. Several previous studies have also suggested that tumour necrosis represent a potential prognostic factor in CRC [22, 26–28, 45]. Still, to our knowledge, our study is the largest so far and also supports the significance of tumour necrosis independent of many parameters currently evaluated in clinical practice such as lymphovascular invasion, tumour grade, MMR status, and *BRAF* status. We compared necrosis percentage and the GMS to establish if necrosis percentage can be used as an additional parameter to supplement the prognostic information of the GMS. We discovered that these parameters represent independent, complementary prognostic factors in CRC. Tumour necrosis percentage has potential to become a predictive biomarker in the future; further studies should assess whether tumours with high necrosis percentage would benefit from specific chemotherapies. For some histological parameters, such as tumour budding in colorectal cancer [46], consensus guidelines have been published to create a clinically valid, reproducible scoring system. Such consensus would also benefit the research of tumour necrosis as a potential cancer biomarker that might guide treatment decisions. Currently, the majority of publications have evaluated overall tumour necrosis percentage, but few studies have compared it to other potential methods of tumour necrosis assessment. Such comparison studies would be valuable to reach a consensus on the optimal evaluated method.

Several limitations of our study must be considered. First, imprecision in the necrosis data might exist, as pathologists usually prefer to sample tumour regions that are not overly necrotic. The necrosis percentage was visually assessed, which may yield some variability in the interpretation. However, these variations are expected to have a nearly random distribution, which would drive our results towards the null hypothesis. Second, tissue microarrays were utilised to analyse immune cell densities, and they are not representative of the overall immune cell infiltrates of the tumours. However, immune cell densities evaluated by tissue microarrays have been shown to be strongly associated with patient survival in CRC [6], supporting the adequacy of this method in capturing clinically meaningful immune cell infiltration patterns. Third, the cross-sectional study design did not enable the examination of the alterations of cytokine concentrations according to necrosis percentage within individual patients. Experimental studies are needed for a more detailed evaluation of the mechanisms linking tumour necrosis and the immune response. Nevertheless, studies involving large human cohorts are valuable, as murine or cell culture models cannot capture the complexity of the human immune system.

In conclusion, tumour necrosis is associated with high circulating CXCL8 concentrations, suggesting that CXCL8 could contribute to the pro-tumour systemic effects of tumour necrosis. Our study supports the value of tumour necrosis as an adverse prognostic factor in CRC independent of disease stage, lymphovascular invasion, tumour grade, MMR status, and *BRAF* status.

## Supplementary information

Supplementary online material

## Data Availability

The datasets generated and/or analyzed during this study are not publicly available. The sharing of data will require approval from relevant ethics committees and/or biobanks. Further information including the procedures to obtain and access data of Finnish Biobanks are described at https://finbb.fi/en/fingenious-service.
