# Supplementary material for: Immunological and prognostic significance of tumour necrosis in colorectal cancer
Source: Br J Cancer. 2023 Apr 8;128(12):2218–26. doi: 10.1038/s41416-023-02258-2 (PMC10241859; doi:10.1038/s41416-023-02258-2)
Supplement: Supplementary file 1 — Supplementary online material [file 41416_2023_2258_MOESM1_ESM.pdf]

Supplementary material

Table S1. Multivariable Cox regression models for cancer-specific survival and overall survival in Cohort 1.

Variable	Cancer-specific survival	Overall survival
	Multivariable	Multivariable
	HR (95% CI)	HR (95% CI)
Necrosis		
< 3%	1 (referent)	1 (referent)
3-9,9%	1.76 (1.02-3.04)	1.19 (0.85-1.66)
10-39.9%	2.35 (1.34-4.11)	1.44 (1.02-2.04)
>40%	3.22 (1.68-6.17)	1.88 (1.19-2.97)
Age		
<65	1 (referent)	1 (referent)
65-75	1.25 (0.92-1.68)	1.39 (1.08-1.78)
>75	2.04 (1.52-2.74)	3.17 (2.51-4.01)
Sex		
Male	1 (referent)	1 (referent)
Female	0.95 (0.74-1.20)	0.77 (0.65-0.93)
Time of operation		
2000 to 2005	1 (referent)	1 (referent)
2006 to 2010	0.62 (0.47-0.83)	0.69 (0.56-0.85)
2011 to 2015	0.48 (0.36-0.65)	0.61 (0.49-0.77)
Tumor location		
Proximal colon	1 (referent)	1 (referent)
Distal colon	0.91 (0.70-1.19)	0.96 (0.79-1.17)
Rectum	0.92 (0.64-1.33)	0.95 (0.72-1.25)
T		
1-2	1 (referent)	1 (referent)
3-4	2.28 (1.39-3.74)	1.31 (1.02-1.71)
N		
0	1 (referent)	1 (referent)
1-2	2.22 (1.67-2.95)	1.40 (1.15-1.71)
M		
0	1 (referent)	1 (referent)
1	7.44 (5.65-9.79)	5.89 (4.67-7.41)
Tumor grade		
Low-grade (well to moderately differentiated)	1 (referent)	1 (referent)
High-grade (poorly differentiated)	1.98 (1.45-2.70)	1.90 (1.50-2.41)
Lymphovascular invasion		
No	1 (referent)	1 (referent)
Yes	1.74 (1.35-2.25)	1.52 (1.23-1.86)
MMR status		
MMR proficient	1 (referent)	1 (referent)
MMR deficient	0.51 (0.31-0.84)	0.65 (0.46-0.92)
BRAF status		
Wild-type	1 (referent)	1 (referent)
Mutant	1.36 (0.90-2.07)	1.46 (1.08-2.00)

Abbreviations: CI, confidence interval; HR, hazard ratio; MMR, mismatch repair.

Table S2 Comparison of the prognostic power of Tumor necrosis percentage and the Glasgow Microenvironment Score (GMS) using Cox regression models for cancer-specific survival.

Variable	No. of cases	No. of events	Model 1 (univariable) HR (95% CI)	Model 2 (multivariable) HR (95% CI)	Model 3 (multivariable) HR (95% CI)
Tumor necrosis percentage					
< 3%	100	15	1 (referent)	1 (referent)	1 (referent)
3-9.9%	577	145	1.73 (1.01-2.94)	1.54 (0.90-2.62)	1.82 (1.05-3.14)
10-39.9%	327	107	2.45 (1.43-4.20)	1.82 (1.06-3.13)	2.35 (1.34-4.11)
≥40%	59	28	4.24 (2.27-7.94)	2.65 (1.41-4.98)	2.87 (1.50-5.51)
p_{trend}			<0.0001	0.0011	0.00023
Glasgow Microenvironment Score					
GMS0	462	54	1 (referent)	1 (referent)	1 (referent)
GMS1	314	93	2.94 (2.10-4.12)	2.82 (2.02-3.96)	1.82 (1.28-2.59)
GMS2	287	148	6.21 (4.54-8.49)	5.75 (4.19-7.89)	2.40 (1.66-3.45)
p_{trend}			<0.0001	<0.0001	<0.0001

The analysis was based on Cohort 1 (N=1,063). The patients who died within 30 days or less after the surgery (N=37) were excluded.

Model 2: Cox proportional hazards regression model that included tumor necrosis percentage and the Glasgow Microenvironment Score.

Model 3: Cox proportional hazards regression model based on Model 2 that was additionally adjusted for age (<65, 65-75, >75), sex (male, female), T (1-2, 3-4), N (0, 1-2), M (0, 1), tumor location (proximal colon, distal colon, rectum), year of operation (2000-2005, 2006-2010, 2011-2015), lymphatic or venous invasion (no, yes), grade (low-grade, high-grade), MMR status (proficient, deficient), and *BRAF* status (wild-type, mutant).

p_{trend} values were calculated by using the four ordinal categories of tumor necrosis percentage and three ordinal categories of the Glasgow Microenvironment Score as continuous variables in univariable and multivariable Cox proportional hazard regression models.

Abbreviations: HR, hazard ratio; CI, confidence interval.

Table S3: Correlations between tumor necrosis percentage and tumor infiltrating immune cells in Cohort 1.

Variable	N (Unadjusted, Adjusted)	Unadjusted		Adjusted	
		Pearson r	p value	Beta	p value
CD3 IM	1042, 1042	-0.071	0.023	-0.052	0.097
CD3 CT	1082, 1082	-0.115	0.0001	-0.099	0.0013
CD8 IM	1052, 1052	-0.075	0.015	-0.059	0.057
CD8 CT	1084, 1084	-0.083	0.006	-0.058	0.060

Abbreviations: IM, invasive margin; CT, center of tumor. The adjusted correlation coefficients (beta) were based on multivariable linear regression models that included age (continuous), sex (male, female), MMR status (proficient, deficient), *BRAF* mutation status (wild-type, mutant), tumor location (colon, rectum) and stage (I-II, III-IV).

Table S4. Tumor and patient characteristics and their association with tumor necrosis in Cohort 2.

			Tumor necrosis percentage				
Characteristic		Total N (%)	< 3%	3–9.9%	10–39.9%	≥40%	p value
Sex	Male	144 (50%)	25 (17%)	62 (43%)	41 (28%)	16 (11%)	0.16
	Female	143 (50%)	36 (25%)	45 (31%)	47 (33%)	15 (10%)	
Age	<65	92 (31%)	23 (25%)	26 (28%)	31 (34%)	12 (13%)	0.23
	65-75	84 (29%)	17 (20%)	40 (48%)	20 (24%)	7 (8.3%)	
	>75	111 (39%)	21 (19%)	41 (37%)	37 (33%)	12 (11%)	
Tumor location	Proximal colon	123 (43%)	33 (27%)	41 (33%)	32 (26%)	17 (14%)	0.061
	Distal colon	73 (25%)	8 (11%)	28 (38%)	30 (41%)	7 (9.6%)	
	Rectum	91 (32%)	20 (22%)	38 (42%)	26 (29%)	7 (7.7%)	
T	T1	15 (5.2%)	6 (40%)	7 (47%)	2 (13%)	0 (0%)	<0.0001
	T2	68 (24%)	25 (37%)	28 (41%)	13 (19%)	2 (2.9%)	
	T3	182 (63%)	28 (15%)	69 (38%)	64 (35%)	21 (12%)	
	T4	22 (7.7%)	2 (9.0%)	3 (14%)	9 (41%)	8 (36%)	
N*	N0	165 (58%)	46 (28%)	65 (39%)	44 (27%)	10 (6.0%)	<0.0001
	N1	78 (27%)	10 (13%)	33 (42%)	26 (33%)	9 (12%)	
	N2	43 (15%)	5 (12%)	9 (21%)	18 (42%)	11 (26%)	
M	M0	246 (86%)	57 (23%)	102 (41%)	65 (26%)	22 (10%)	<0.0001
	M1	41 (14%)	4 (9.8%)	5 (12%)	23 (56%)	9 (22%)	
Stage*	I	66 (23%)	27 (41%)	27 (41%)	11 (17%)	1 (1.5%)	<0.0001
	II	93 (33%)	18 (19%)	37 (40%)	30 (32%)	8 (8.6%)	
	III	86 (30%)	12 (14%)	38 (44%)	24 (28%)	12 (14%)	
	IV	41 (14%)	4 (9.8%)	5 (12%)	23 (56%)	9 (22%)	
WHO grade	Low-grade	251 (88%)	52 (21%)	100 (40%)	78 (31%)	21 (8.4%)	0.002
	High-grade	36 (13%)	9 (25%)	7 (19%)	10 (28%)	10 (28%)	
MMR enzyme status	Proficient	247 (86%)	42 (17%)	93 (38%)	84 (34%)	28 (11%)	<0.0001
	Deficient	40 (14%)	19 (48%)	14 (35%)	4 (10%)	3 (7.5%)	
BRAF status	Wild-type	105 (90%)	13 (12%)	38 (36%)	41 (39%)	13 (12%)	0.27
	Mutant	12 (10%)	3 (25%)	4 (33%)	2 (17%)	3 (25%)	

* Data missing from one patient

Table S5. Multivariable Cox regression models for cancer-specific survival and overall survival in Cohort 2.

Variable	Cancer-specific survival	Overall survival
	Multivariable HR (95% CI)	Multivariable HR (95% CI)
Necrosis		
< 3%	1 (referent)	1 (referent)
3-9,9%	1.80 (0.71-4.60)	1.08 (0.62-1.88)
10-39.9%	1.85 (0.75-4.54)	1.10 (0.62-1.96)
>40%	3.39 (1.04-8.96)	1.70 (0.86-3.36)
Age		
<65	1 (referent)	1 (referent)
65-75	1.88 (1.03-3.42)	1.96 (1.17-3.30)
>75	2.21 (1.16-4.19)	4.83 (2.94-7.94)
Sex		
Male	1 (referent)	1 (referent)
Female	1.36 (0.83-2.21)	1.00 (0.69-1.44)
Year of operation		
2006 to Jan 2010	1 (referent)	1 (referent)
Feb 2010 to 2014	0.65 (0.39-1.09)	0.69 (0.47-1.02)
Tumor location		
Proximal colon	1 (referent)	1 (referent)
Distal colon	1.24 (0.66-2.33)	1.12 (0.69-1.84)
Rectum	0.94 (0.52-1.73)	1.06 (0.67-1.67)
T		
1-2	1 (referent)	1 (referent)
3-4	3.60 (1.63-7.91)	1.44 (0.89-2.33)
N		
0	1 (referent)	1 (referent)
1-2	3.39 (1.78-6.44)	1.78 (1.15-2.75)
M		
0	1 (referent)	1 (referent)
1	8.59 (4.71-15.67)	6.39 (3.93-10.40)
Tumor grade		
Low-grade (well to moderately differentiated)	1 (referent)	1 (referent)
High-grade (poorly differentiated)	1.50 (0.78-2.92)	1.02 (0.60-1.74)
Lymphovascular invasion		
No	1 (referent)	1 (referent)
Yes	1.55 (0.79-3.05)	1.14 (0.72-1.79)
MMR status		
MMR proficient	1 (referent)	1 (referent)
MMR deficient	0.16 (0.02-1.25)	0.83 (0.41-1.69)
BRAF status		
Wild-type	1 (referent)	1 (referent)
Mutant	2.02 (0.77-5.33)	1.41 (0.70-2.81)

Abbreviations: CI, confidence interval; HR, hazard ratio; MMR, mismatch repair.

Table S6. Correlations between tumor necrosis percentage and tumor infiltrating immune cells in Cohort 2.

Variable	N (Unadjusted, Adjusted)	Unadjusted		Adjusted	
		Pearson r	p value	Beta	p value
CD3 IM	287, 286	-0.104	0.078	-0.009	0.88
CD3 CT	287, 286	-0.167	0.004	-0.071	0.23
CD3 IEL	287, 286	-0.157	0.008	-0.056	0.36
CD8 IM	287, 286	-0.131	0.026	-0.050	0.40
CD8 CT	287, 286	-0.193	0.001	-0.100	0.096
CD8 IEL	287, 286	-0.122	0.038	-0.024	0.69
FoxP3 IM	286, 285	-0.233	<0.001	-0.113	0.067
FoxP3 CT	286, 285	-0.208	<0.001	-0.109	0.068
Mast cell IM	286, 285	-0.274	<0.001	-0.196	0.001
Mast cell CT	286, 285	-0.079	0.18	-0.034	0.55
Neutrophil IM	282, 281	-0.065	0.27	0.018	0.76
Neutrophil CT	282, 281	-0.039	0.51	0.033	0.56

The adjusted correlation coefficients (beta) were based on multivariable linear regression models that included age (continuous), sex (male, female), tumor location (colon, rectum), stage (I-II, III-IV), MMR enzyme status (proficient, deficient), and *BRAF* status (wild-type, mutant).
Abbreviations: IM, invasive margin; CT, center of tumor; IEL, intraepithelial

Table S7. Associations between mismatch repair (MMR) status and immune cells in Cohort 2.

	N (Proficient, Deficient)	MMR proficient, Median (IQR)	MMR deficient, Median (IQR)	p value
CD3 IM	247,40	561 (323-800)	787 (578-1170)	<0.0001
CD3 CT	247,40	399 (217-651)	699 (251-1150)	0.0033
CD3 IEL	247,40	18.8 (6.06-57.7)	60.3 (18.9-267)	<0.0001
CD8 IM	247,40	160 (84.8-319)	335 (139-681)	<0.0001
CD8 CT	247,40	89.6 (31.6-198)	273 (68.4-591)	0.0002
CD8 IEL	247,40	12.5 (3.29-44.2)	46.5 (16.9-206)	<0.0001
FoxP3 IM	246,40	166 (84.3-254)	300 (116-410)	0.0001
FoxP3 CT	246,40	134 (65.0-257)	170 (75.6-383)	0.080
Mast IM	246,40	40.6 (26.2-74.0)	57.5 (26.4-104)	0.043
Mast CT	246,40	28.4 (14.8-54.3)	33.8 (21.1-55.2)	0.356
Neutrophil IM	242,40	38.3 (10.0-129)	96.1 (18.5-269)	0.041
Neutrophil CT	242,40	24.4 (6.67-87.2)	66.4 (13.3-169)	0.005

Abbreviations: IM, invasive margin; CT, center of tumor; IEL, intraepithelial; IQR, interquartile range

Table S8. Tumor and patient characteristics in Cohort 3 and their association with tumor necrosis.

Characteristic		Total N(%)	Tumor necrosis percentage < 3%	3–9.9%	10–39.9%	≥40%	p value
Sex	Male	14 (54%)	3 (21%)	4 (29%)	6 (43%)	1 (7.1%)	0.56
	Female	12 (46%)	3 (25%)	6 (50%)	3 (25%)	0 (0%)	
Age	≤65	5 (19%)	2 (40%)	2 (40%)	1 (20%)	0 (0%)	0.75
	>65	21 (81)	4 (19%)	8 (38 %)	8 (38%)	1 (4.8%)	
Tumor location	Proximal colon	18 (69%)	6 (33%)	7 (39%)	4 (22%)	1 (5.6%)	
	Distal colon	8 (31%)	0 (0%)	3 (38%)	5 (63%)	0 (0%)	
T	T1	2 (7.7%)	0 (0%)	2 (100%)	0 (0%)	0 (0%)	0.36
	T2	9 (35%)	3 (33%)	4 (44%)	2 (22%)	0 (0%)	
	T3	10 (39%)	2 (20%)	2 (20%)	6 (60%)	0 (0%)	
	T4	5 (19%)	1 (20%)	2 (40%)	1 (20%)	1 (20%)	
N	N0	17 (65%)	3 (18%)	7 (41%)	7 (41%)	0 (0%)	0.30
	N1	4 (15%)	1 (25%)	2 (50%)	0 (0%)	1 (25%)	
	N2	5 (19%)	2 (40%)	1 (20%)	2 (40%)	0 (0%)	
M	M0	26 (100%)	6 (23%)	10 (38%)	9 (35%)	1 (3.8%)	
Stage	I	10 (39%)	2 (20%)	6 (60%)	2 (20%)	0 (0%)	0.24
	II	7 (27%)	1 (14%)	1 (14%)	5 (71%)	0 (0%)	
	III	9 (35%)	3 (33%)	3 (33%)	2 (22%)	1 (11%)	
WHO grade	Low-grade	18 (69%)	2 (11%)	9 (50%)	7 (39%)	0 (0%)	0.039
	High-grade	8 (31%)	4 (50%)	1 (13%)	2 (25%)	1 (13%)	
